# Mesenchymal Stem Cell–Sourced Exosomes as Potentially Novel Remedies for Severe Dry Eye Disease

**DOI:** 10.1155/joph/5552374

**Published:** 2025-01-10

**Authors:** Carl Randall Harrell, Valentin Djonov, Ana Volarevic, Aleksandar Arsenijevic, Vladislav Volarevic

**Affiliations:** ^1^Department of Molecular Biology, Regenerative Processing Plant, LLC, 34176 US Highway 19 N, Palm Harbor, Florida, USA; ^2^Department of Anatomy, Institute of Anatomy, University of Bern, Baltzerstrasse 2, Bern 3012, Switzerland; ^3^Departments of Psychology, Center for Research on Harmful Effects of Biological and Chemical Hazards, Faculty of Medical Sciences University of Kragujevac, 69 Svetozara Markovica Street, Kragujevac 34000, Serbia; ^4^Departments of Genetics, Microbiology and Immunology, Center for Research on Harmful Effects of Biological and Chemical Hazards, Faculty of Medical Sciences University of Kragujevac, 69 Svetozara Markovica Street, Kragujevac 34000, Serbia; ^5^Department of Biology, Faculty of Pharmacy Novi Sad, Trg Mladenaca 5, Novi Sad 21000, Serbia

**Keywords:** dry eye disease, exosomes, immunomodulation, mesenchymal stem cells, therapy

## Abstract

Severe dry eye disease (DED) is an inflammatory condition characterized by a lack of sufficient moisture or lubrication on the surface of the eye, significantly impacting the quality of life and visual function. Since detrimental immune response is crucially responsible for the development and aggravation of DED, therapeutic agents which modulate phenotype and function of eye-infiltrated inflammatory immune cells could be used for the treatment of severe DED. Due to their potent immunomodulatory properties, mesenchymal stem cells (MSCs) represent potentially new remedies for the treatment of inflammatory eye diseases. The majority of MSC-sourced bioactive factors are contained within MSC-derived exosomes (MSC-Exos), nano-sized extracellular vesicles which, due to their nanosize dimension and lipid envelope, easily by pass all biological barriers in the body and deliver their cargo directly into the target immune cells. MSC-Exos contain a variety of bioactive proteins (growth factors, immunoregulatory molecules, cytokines, and chemokines) lipids, and microRNAs (miRNAs) which affect viability, proliferation, phenotype, and function of eye-infiltrated immune cells. Accordingly, MSC-Exos may modulate the progression of inflammatory eye diseases, including DED. Therefore, in this review article, we summarized the current knowledge regarding molecular and cellular mechanisms which were responsible for trophic, anti-inflammatory, immunoregulatory, and regenerative properties of MSC-Exos in the treatment of severe DED. For this purpose, an extensive literature review was carried out in February 2024 across several databases (Medline, Embase, and Google Scholar), from 2000 to the present. Eligible studies delineated molecular and cellular mechanisms responsible for the MSC-Exos–based modulation of immune cell–driven eye inflammation in DED, and their findings were analyzed in this review. Results obtained in these studies demonstrated beneficial effects of MSC-Exos in the treatment of severe DED, paving the way for their future clinical use in ophthalmology.

**Trial Registration:** ClinicalTrials.gov identifier: NCT04213248, NCT06475027, NCT06543667, NCT05738629

## 1. Introduction

Dry eye disease (DED), also referred to as dry eye syndrome or keratoconjunctivitis sicca, is an inflammatory condition characterized by inadequate moisture or lubrication on the eye's surface [1]. The prevalence of DED ranges from 5% to 50%, depending on the geographic region, with the highest in Africa and the lowest prevalence in North America. DED is more frequently diagnosed in Asian than in Caucasian populations [2]. DED is a debilitating disease occurring in all ages, with increased frequency in older patients. The prevalence of DED starts at 2.7% for individuals aged 18–34 and reaching 18.6% for those 75 years and older. In the 18–34 age group, 2.9% of women and 2.6% of men were diagnosed with DED, while in the 75 years and older group, the rates increased to 22.8% for women and 12.6% for men [2]. The prevalence of DED in children ranges from 5.5% to 23.1% [3]. Although DED can occur in healthy individuals, it is more commonly seen in patients with autoimmune disorders, such as Sjögren's syndrome or rheumatoid arthritis [2]. Similarly, DED is more frequently diagnosed in children with previous history of ocular allergy or systemic inflammatory diseases [3]. It should be emphasized that people who wear contact lenses, especially for extended periods, often experience symptoms of DED [4]. Individuals who work in environments with low humidity, such as air-conditioned offices, or who spend long hours in front of screens are also at higher risk to develop DED [4]. Severe DED, an advanced form of the condition, is marked by substantial and persistent symptoms that significantly affect the individual's quality of life and visual function [5]. It is a long-term ailment that results in an unhealthy tear film, leading to intense dryness, discomfort, and potential harm to the eye's surface [5]. Individuals with severe DED commonly endure continual eye discomfort, a persistent burning or stinging sensation, and redness or bloodshot appearance due to inflammation and irritation from insufficient lubrication [6]. The eyes may feel excessively dry, gritty, or sandy, and severe DED can lead to vision issues such as blurred or unstable vision [6].

The insufficiency of lubrication can result in persistent inflammation [7–9]. Prolonged and severe dryness of the eye surface can cause damage to the cornea and induce formation of corneal erosions, ulcers, or thinning [7]. When damaged associated molecular patterns (DAMPs) and alarmins are released from injured corneal and conjunctival epithelial cells (ECs), they activate professional antigen-presenting cells such as dendritic cells (DCs) and macrophages [6–8]. These cells produce inflammatory cytokines such as tumor necrosis factor-alpha (TNF-*α*) and interleukin 1 beta (IL-1*β*), leading to an increased expression of E and P selectins on endothelial cells (ECs) [6]. This enables a significant influx of circulating leukocytes into the inflamed eyes of patients with DED [4]. Among the recruited inflammatory immune cells, CD4+, Th1, and Th17 lymphocytes that produce interferon-gamma (IFN-*γ*) and IL-17 play a crucial pathogenic role in the development and progression of severe DED [6–8]. IFN-*γ* from Th1 cells induces the synthesis of inflammatory mediators such as nitric oxide (NO), reactive oxygen species (ROS), TNF-*α*, IL-1*β*, IL-6, IL-12, and IL-23, promoting the generation of an inflammatory phenotype in eye-infiltrated monocytes/macrophages [6–8]. IL-17 from Th17 cells stimulates the production of ROS, NO, TNF-*α*, and IL-1*β* in neutrophils, enhancing neutrophil extracellular trap (NET) formation, which significantly contributes to the ongoing eye inflammation [7]. In addition to CD4+ T helper cells, CD8+ cytotoxic lymphocytes (CTLs) also infiltrate the lacrimal glands (LGs) of individuals with severe DED [6]. CTLs release TNF-*α*, perforins, and granzymes, leading to the apoptosis of secretory cells in the LGs, reducing tear production and worsening the dryness and discomfort of the eyes [6–8]. Furthermore, chronic T-cell–driven inflammation can harm the ocular surface barrier, which is essential for maintaining ocular surface health [6, 7]. The damage of corneal and conjunctival ECs can compromise barrier function, increase tear evaporation, and further worsen DED-related symptoms [6–8].

Conventional treatments such as adding tears and using lubricating eye drops can help relieve symptoms and support the healing of the eye surface in individuals with DED [10]. However, adjusting the characteristics and functions of immune cells is crucial in managing severe DED and enhancing the overall eye health of those affected [11]. Therefore, gaining a better understanding of the molecular mechanisms that coordinate harmful immune responses in severe DED patients is vital for developing specific treatment strategies [10, 11]. Anti-inflammatory drugs such as corticosteroids or immunomodulatory agents may suppress inflammation driven by immune cells and potentially alleviate DED symptoms [11]. While corticosteroids and immunosuppressants can effectively manage severe DED, their prolonged use may have adverse effects, such as increased vulnerability to infections and reduced ability to repair and regenerate the damaged eye surface [11, 12]. Furthermore, extended use of steroids could lead to conditions such as glaucoma, cataracts, and corneal thinning in DED patients [11, 12]. In addition, these immunomodulatory medications may interact with other drugs, causing potential side effects [11–13].

It is important to note that the eye drops used in severe DED treatment do not contain growth factors and cannot promote the healing and regeneration of damaged cells in the LGs or eye surface of DED patients [11]. Moreover, the availability and long-term impact of eye drops are generally limited due to the eye's efficient mechanisms that quickly clear them from the precorneal area [11, 12]. Therefore, there is an urgent need for new immunomodulatory agents that can reduce ongoing eye inflammation without compromising the protective immune response and can aid in the regeneration of damaged epithelial barriers and LGs in severe DED patients [13].

Due to their capacity to produce large number of immunomodulatory factors which may efficiently regulate detrimental immune response in the eyes and may attenuate systemic inflammation without causing any severe adverse effects, mesenchymal stem cells (MSCs) and their secretome were considered as potentially novel therapeutic agents in the treatment of inflammatory eye diseases, including severe DED [14].

## 2. MSC-Derived Exosomes: Potentially New Remedies in Ophthalmology

MSCs are mature, self-renewable, spindle-shaped cells that can be easily isolated from several tissues [15–17]. The most common source is bone marrow, where they reside within the stromal niche, a microenvironment that supports their maintenance and function. In addition to bone marrow, MSCs can also be harvested from adipose tissue (AT), dental pulp (DP), umbilical cord (UC), amniotic fluid (AF), and even LGs [16, 17]. AT-derived MSCs (AT-MSCs) can be harvested through minimally invasive liposuction procedures, making them more patient-friendly [16]. These cells produce a large number of immunomodulatory and growth factors and may efficiently suppress detrimental immune response in inflamed eyes, importantly contributing to the attenuation of ongoing inflammation [17]. DP is another exceptional source of MSCs [16]. Found within the central cavity of teeth, DP contains a rich supply of DP-derived MSCs (DP-MSCs) that can differentiate into odontoblasts, which are crucial for dentin formation, osteocytes, and even in neuron-like cells [16, 17]. One specific advantage of DP-MSCs is their ability to produce growth factors, including bone morphogenetic proteins (BMPs) which can promote the healing of critical-sized bone defects [17]. Furthermore, an easy accessibility from extracted teeth, particularly in children and young adults, makes DP-MSC a promising and ethically favorable option for cell-based regenerative therapies [16, 17]. UC tissue, particularly Wharton's jelly, is a highly promising source of MSCs due to its unique properties and noninvasive collection method [16]. UCs are typically discarded after birth, providing an ethically sound and abundant source of MSCs without the ethical concerns. UC-derived MSCs (UC-MSCs) exhibit a remarkable capacity for self-renewal, multilineage differentiation, and immunomodulation [16, 17]. Therefore, UC-MSCs may be used in cell-based therapy of inflammatory and autoimmune diseases [15]. Similarly, AF-derived MSCs (AF-MSCs) also represent potentially novel therapeutic agents in regenerative medicine [15]. AF-MSCs, harvested during amniocentesis, exhibit a high degree of plasticity and may differentiate into multiple cell lineages, including neural and cardiac cells [16].

LGs present a fascinating and underexplored source of MSCs with unique characteristics that make them valuable for regenerative medicine [16]. One specificity of LG-derived MSCs (LG-MSCs) is their ability to secrete neurotrophic factors, which can support neuronal survival and enhance regeneration in ocular tissues, making them particularly promising for treating conditions such as severe DED and nerve injuries [18]. In addition, LG-MSCs produce different immune-regulating substances to suppress harmful immune reactions in the inflamed eyes [18].

MSCs from all tissues have potential to modulate phenotype and functions of immune cells that contribute to the development and progression of severe DED [19–21]. Upon the injury, MSCs are recruited to the sites of tissue damage and are activated by local inflammatory cytokines produced by tissue resident immune cells [22]. If MSCs are exposed to the higher concentration of immunosuppressive cytokines, MSCs may obtain a proinflammatory phenotype [22]. These “proinflammatory” MSCs produce inflammatory cytokines that could promote generation and expansion of inflammatory immune cells in secondary lymph organs [22]. However, when MSCs engraft in an inflamed microenvironment, they become exposed to the high concentration of inflammatory cytokines. Under the influence of TNF-*α* and IFN-*γ*, MSCs obtain an “immunosuppressive phenotype” and produce large number of anti-inflammatory cytokines that suppress proliferation and activation of inflammatory immune cells [22]. MSCs can reduce ongoing eye inflammation by altering macrophage activation, inducing a tolerogenic state in DCs, and decreasing the production of harmful substances in infiltrated neutrophils [15, 21, 22]. MSCs can also inhibit the expression of molecules that promote immune responses and suppress the production of cytokines associated with Th1 and Th17 immune responses, thereby reducing antigen presentation by macrophages and DCs and preventing the expansion of harmful immune cells that drive eye inflammation [15].

While MSCs offer significant therapeutic potential, their clinical use in DED treatment is limited due to various side effects resulting from their transplantation [23]. Despite having low levels of major histocompatibility (MHC) Class II molecules, MSCs are not immune-privileged cells, and their transplantation can trigger harmful immune responses due to the recognition of foreign molecules by the recipient's immune system [23]. This can lead to rejection of the transplanted cells and immune cell–driven inflammation [23]. Another potential side effect of MSC transplantation is their unintended differentiation into cell types such as chondrocytes and osteocytes, which can disrupt tissue structure and function [23]. Although rare, there have been instances where MSCs have been linked to tumor formation or the promotion of existing tumors [20, 24]. This risk is associated with the ability of MSCs to differentiate into various cell types, including those involved in cancer development [20, 24]. MSCs release proangiogenic factors (angiopoietin, vascular endothelial growth factor [VEGF], and IL-6) that support the growth of new blood vessels in the tumor environment, potentially aiding in the spread of malignant cells [24].

The therapeutic benefits of MSCs mainly rely on the biological activity of MSC-sourced growth factors, proangiogenic and immunomodulatory factors [21]. Therefore, injection of MSC-derived secretome is considered as a novel approach for the treatment of inflammatory diseases since it can address all safety concerns associated with transplantation of MSCs [21]. The majority of MSC-sourced bioactive compounds are found in MSC-derived exosomes (MSC-Exos), small extracellular vesicles (EVs) abundantly present in the secretome of MSCs [25]. MSC-Exos are uniform in nature, display surface markers typical of their parent cells (such as CD9, CD63, CD81, or CD44), and exhibit a consistent size and shape [25]. These exosomes are characterized by their small size (30–150 nm), rounded or cup-shaped structure, and lipid bilayer membrane, which facilitate their vital role in cellular communication [26]. The outer membrane of MSC-Exos is composed of phospholipids, cholesterol, and glycolipids. Because of their diminutive size and lipid envelope, MSC-Exos can easily traverse biological barriers in the body and deliver their cargo directly to target cells [26]. MSC-Exos contain a diverse array of bioactive molecules, including proteins (growth factors, immunoregulatory substances, cytokines, and chemokines), lipids, and nucleic acids (such as messenger RNA and microRNAs), which can impact the viability, growth, characteristics, and functions of both parenchymal and immune cells in damaged and inflamed tissues [25, 26]. Recent experimental studies have showed the beneficial effects of MSC-Exos in managing severe inflammatory eye disorders, indicating their potential for therapeutic use in clinical settings [25–27]. Accordingly, in this review article, we summarized the current knowledge regarding molecular and cellular mechanisms which were responsible for trophic, anti-inflammatory, immunoregulatory, and regenerative properties of MSC-Exos in the treatment of severe DED. An extensive literature review was carried out in February 2024 across several databases (Medline, Embase, and Google Scholar), from 2000 to present. Keywords used in the selection were as follows: “mesenchymal stem cells,” “exosomes,” “dry eye disease,” “eye inflammation,” “miRNAs,” “signaling pathways,” “viability,” “apoptosis,” “proliferation,” “differentiation,” “immunomodulation,” and “tissue repair and regeneration.” All journals were considered, and initial search retrieved 175 articles. The abstracts of all these articles were subsequently reviewed by two of the authors (CRH and VV) independently to check their relevance to the subject of this manuscript. Among them, 23 studies delineated molecular and cellular mechanisms responsible for the MSC-Exos–based modulation of immune cell–driven eye inflammation in DED, and their findings were analyzed in detail in this review.

## 3. MSC-Exos–Based Attenuation of Innate Immune Cell–Driven Eye Inflammation in DED

Wang et al. used murine model of benzalkonium chloride (BAC)–induced DED to examine therapeutic efficacy of MSC-Exos in the suppression of eye inflammation [28]. Two weeks after topical administration of 0.2% BAC, mice were divided into experimental and control groups randomly to receive either MSC-Exos, commercial eye drops containing 0.1% pranoprofen, or phosphate-buffered saline (PBS). Each treatment was given three times for 7 days [28]. MSC-Exos suppressed ongoing eye inflammation in a dose-dependent manner. The most remarkable MSC-Exos–dependent ocular surface epithelial repair was observed in the group of BAC-treated mice that received 50 mg/mL of MSC-Exos [28]. MSC-Exos enhanced tear film stability and prevented inflammation-induced apoptosis of corneal ECs (CECs) [28, 29]. BAC led to a significant release of alarmins from injured CECs. These alarmins activated the nucleotide-binding domain, leucine-rich–containing family, and pyrin domain–containing-3 (NLRP3) inflammasome in eye-infiltrated neutrophils and macrophages, resulting in increased production of inflammatory cytokines IL-1*β*, IL-6, IL-1*α*, and TNF-*α* [29]. The elevated levels of these mediators led to enhanced recruitment of circulating leukocytes in the inflamed eyes of BAC-treated animals [29]. Wang et al. noted significantly lower levels of inflammatory cytokines (IL-1*β*, IL-6, IL-1*α*, TNF-*α*, and IFN-*γ*) in the serum samples of MSC-Exos–treated animals compared to PBS-treated control animals, indicating the suppressive effects of MSC-Exos on the systemic immune response [28]. Importantly, MSC-Exos enhanced the production of immunosuppressive IL-10, which suppressed the generation of an inflammatory phenotype in eye-infiltrated neutrophils and monocytes. Consequently, reduced expression of NLRP3, IL-1*β*, and IL-18 was observed in conjunctival tissue samples of BAC + MSC-Exos–treated animals compared to BAC-treated mice, indicating that the MSC-Exos–mediated inhibition of NLRP3 inflammasome in eye-infiltrated immune cells was mainly responsible for the beneficial effects of MSC-Exos in DED treatment [28]. MSC-Exo–based attenuation of NLRP3-driven inflammation suppressed activation of Caspase-3 in CECs. The MSC-Exos–dependent inhibition of Caspase-3–driven apoptosis prevented the increased loss of BAC-injured CECs and facilitated the enhanced regeneration of the ocular surface epithelial barrier [28].

**Figure 1:**
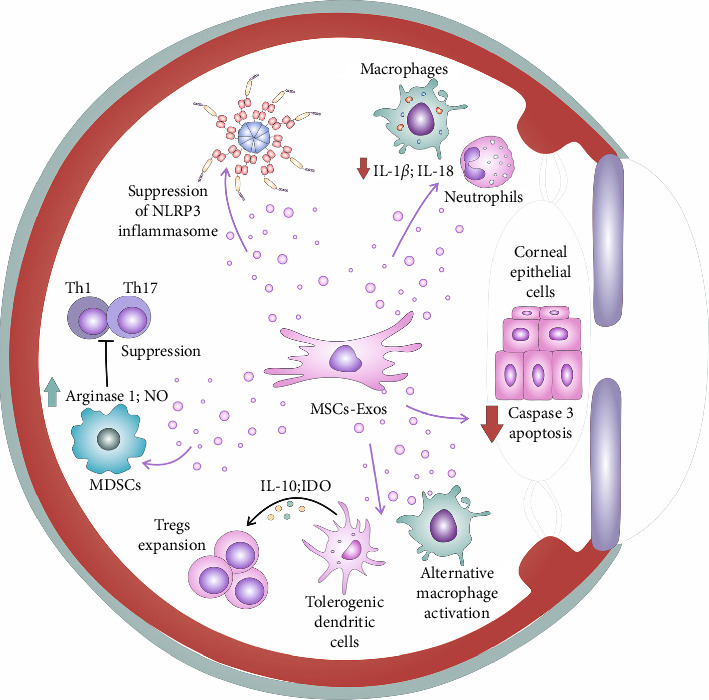
Mesenchymal stem cell–derived exosomes (MSC-Exos)–based suppression of detrimental immune response in the inflamed eyes. MSC-Exos suppress expression of nucleotide-binding domain, leucine-rich–containing family, pyrin domain–containing-3 (NLRP3), and interleukin (IL)-1*β* and IL-18, attenuating generation of inflammatory phenotype in eye-infiltrated neutrophils and monocytes. MSC-Exo–based attenuation of NLRP3-driven inflammation results in the suppression of Caspase 3 and prevents loss of corneal epithelial cells. By suppressing activation of NLRP3 inflammasome, MSC-Exos–induced generation of alternatively activated (M2) phenotype in eye-infiltrated macrophages M2 macrophages interact with other eye-infiltrated anti-inflammatory cells (tolerogenic dendritic cells (DCs) and CD4+ CD25+ T–regulatory cells (Tregs)) and create immunosuppressive microenvironment in inflamed eyes, crucially contributing to the attenuation of eye inflammation and to the alleviation of dry eye disease (DED)–related symptoms. Tolerogenic DCs produce IL-10 and indoleamine 2, 3-dioxygenase (IDO) which promotes generation and expansion of immunosuppressive Tregs in inflamed tissues. Also, MSC-Exos increased expression of immunoregulatory factors (arginase-1 and nitric oxide [NO]) in myeloid-derived suppressor cells (MDSCs) which can inhibit the proliferation of activated Th1 and Th17 cells.

By inhibiting the activation of the NLRP3 inflammasome, MSC-Exos stimulated the development of alternatively activated (M2) phenotype in eye-infiltrated macrophages [30]. These M2 macrophages interact with other anti-inflammatory cells (tolerogenic DCs and forkhead box P3 (FoxP3)–expressing CD4+CD25 + T–regulatory cells (Tregs)), to establish an immunosuppressive environment in the inflamed eyes (Figure 1) [30]. This cross-talk between M2 macrophages, tolerogenic DCs, and Tregs in inflamed eyes plays a crucial role in reducing ongoing inflammation and in alleviating DED-related symptoms [30]. M2 macrophage–derived immunosuppressive transforming growth factor beta (TGF-*β*) and MSC-Exos–sourced IL-10 inhibit the maturation of DCs and promote their differentiation into tolerogenic DCs [21]. Tolerogenic DCs exhibit suboptimal expression of MHC Class II and costimulatory molecules and instead of producing pro-Th1 and pro-Th17 cytokines, they secrete IL-10 and indolamine 2,3-dioxygenase (IDO) to support the generation and expansion of immunosuppressive Tregs in inflamed tissues [30]. IDO, which is produced by both MSCs and tolerogenic DCs, metabolizes tryptophan (TRP) to generate kynurenine, leading to increased expression of the FoxP3 transcription factor in naïve CD4+ T lymphocytes. This process enables the expansion of immunosuppressive Tregs in the inflamed eyes, resulting in the attenuation of ongoing inflammation [21].

Zhou et al. used the murine model of BAC-induced ocular graft versus disease (oGVHD) to demonstrate that MSC-Exos efficiently suppressed eye inflammation by inducing expansion of immunosuppressive M2 macrophages in the eyes of experimental mice [31]. By delivering immunoregulatory miR-204, MSC-Exos reprogramed inflammatory M1 macrophages into immunosuppressive M2 cells and alleviated M1 macrophage–driven inflammation [31]. MSC-Exos–sourced miR-204 modulated IL-6/IL-6R/Stat3 pathway in eye-infiltrated macrophages, suppressed IL-6-driven eye inflammation, and attenuated DED-related symptoms in experimental animals [31]. Similar results were reported by Ren et al. who showed that MSC-Exos induced generation of immunosuppressive M2 phenotype in macrophages, enhanced production of anti-inflammatory IL-10 and arginase-1, and protected conjunctival goblet cells from macrophage-driven injury [32].

Apart from M2 macrophages, tolerogenic DCs, and Tregs, myeloid-derived suppressor cells (MDSCs) also generate anti-inflammatory cytokines such as TGF-*β* and IL-10, playing a key role in dampening harmful immune responses in the inflamed eyes of individuals with DED [33]. MDSCs also release immunoregulatory factors such as Arginase-1 and NO, which can hinder the proliferation of activated Th1 and Th17 cells [34]. MDSCs-derived NO hinders the cell cycle progression by inhibiting cyclin-dependent kinases and induces apoptosis in inflammatory T cells through the activation of Caspase-3 [33, 34]. In addition, MDSC-sourced NO reduces the expression of IL-2, a critical factor responsible for the expansion of activated T cells [33, 34]. Arginase-1, another immunoregulatory factor produced by MDSCs, works by metabolizing the amino acid arginine [33]. By depleting arginine, MDSCs impede the growth of effector Th1 and Th17 cells [33, 34]. Furthermore, MDSC-derived Arginase-1 can redirect arginine metabolism toward the production of polyamines and proline, which are involved in tissue repair, thereby aiding in the restoration of damaged ocular surface barriers [33]. Recently, Rui et al. used a murine model of primary Sjögren's syndrome (pSS) to evaluate the therapeutic potential of MSC-Exos which were derived from murine olfactory ecto-MSCs (OE-MSC-Exos) [35]. They showed that OE-MSC-Exos improved the immunosuppressive capabilities of eye-infiltrated MDSCs and alleviated DED-related symptoms in experimental animals. The intravenous infusion of OE-MSC-Exos (100 *μ*g) induced the generation of Arginase-1 and NO while reducing the levels of MHC Class II and costimulatory molecules (CD40, CD80, and CD86) in MDSCs, leading to the inhibition of T-cell–induced eye inflammation [35]. OE-MSC-Exo–derived S100A4, a member of the S100 calcium-binding protein family and ligand of toll-like receptor (TLR)-4, had a significant impact on the immunosuppressive properties of OE-MSC-Exos [35]. By activating TLR-4 signaling, OE-MSC-Exos modulated the Janus kinase (Jak)2/signal transducers and activators of transcription (Stat)3 pathway in MDSCs, increasing IL-6 production. MDSC-sourced IL-6, then, in autocrine, paracrine, and endocrine manner, boosts the expression of Arginase-1 and NO in eye-infiltrated MDSCs, crucially aiding in the suppression of Th1 and Th17 cell-driven injury and inflammation in the eyes of OE-MSC-Exos–treated mice, alleviating DED-related symptoms [35].

## 4. Molecular Mechanisms Responsible for MSC-Exo–Based Modulation of T-Cell–Driven Eye Inflammation

Ongoing eye inflammation and antigen-dependent priming of T-cell receptors results in the phosphorylation of protein kinase B (PKB/Akt) and mammalian target of rapamycin (mTOR) in resting Tregs [7]. Activation of the Akt/mTOR pathways causes a change in the immunoregulatory characteristics of Tregs, leading to their transformation into a proinflammatory Th17-like phenotype. This altered phenotype is characterized by increased production of inflammatory cytokines, particularly IL-17 and IL-22. By reducing the TRP levels in the inflamed environment, MSC-Exos–derived IDO triggers the activation of the general control non-derepressible 2 (GCN2) kinase, which inhibits the Akt/mTOR2 signaling in Tregs and prevents their conversion into inflammatory Th17 cells. Therefore, MSC-Exos, in an IDO-dependent manner, suppress generation of Th17 cells and facilitate the expansion of immunosuppressive Tregs in the inflamed eyes of DED patients, contributing to the Tregs-dependent alleviation of Th17 cell-driven inflammation [24]. Zhao et al. demonstrated that MSC-Exos–derived miR-21-5p enhanced Tregs-driven suppression of inflammatory Th17 cells in the eyes of DED experimental mice, alleviating DED-related symptoms. MSC-Exos–sourced miR-21-5p decreased production of inflammatory cytokines (IL-17 and IL-22) and promoted synthesis of immunosuppressive cytokines (IL-10 and TGF-*β*1) in eye-infiltrated T cells. Importantly, MSC-Exos, through the delivery of miR-21-5p, increased the total number of goblet cells, improved tear secretion, and significantly attenuated DED-related symptoms in experimental animals, suggesting that upregulation of miR-21-5p in MSC-Exos should be explored as potentially novel approach for enhancement of MSC-Exos' efficacy in DED treatment [36].

In line with these findings are results recently obtained by Li et al. and Ma et al. who confirmed that beneficial effects of MSC-Exos in the attenuation of DED-related symptoms were mainly relied on MSC-Exos–dependent enhancement of Treg-driven immunosuppression of inflammatory Th1 and Th17 cells [37, 38]. Li et al. isolated MSC-Exos from human labial glands (LG-MSC-Exos) and evaluated their immunoregulatory effects in experimental animals and in vitro, by analyzing MSC-Exos–dependent changes of mononuclear cells (MNCs) previously isolated from the blood of pSS patients [37]. The hallmark characteristic of this autoimmune disorder is dryness of the eyes due to immune cell–mediated destruction of LGs [39]. Th1 and Th17 cells, through the activity of IFN-*γ* and IL-17, contribute to the apoptosis of epithelial and acinar cells and trigger a robust systemic inflammatory response that leads to the production of auto-antibodies targeting the self-antigens of LGs [39]. Th1 cell–sourced IFN-*γ* induces apoptosis of salivary gland ECs (SGECs) and importantly contributes to the development of pSS-related symptoms [40]. Hu et al. demonstrated that MSC-Exos efficiently attenuated detrimental effects of IFN-*γ* on SGECs [40]. MSC-Exos enhanced the expression of aquaporin 5 (AQP5) on SGECs, activated protein kinase A/cyclic-AMP response element binding (PKA/CREB) pathway, revitalized function of salivary glands, and alleviated DED-related symptoms in experimental mice [40].

The imbalance of effector Th1 and Th17 lymphocytes with reduced immunosuppressive Tregs establishes a cycle of inflammation in the eyes of pSS patients, ultimately causing chronic eye inflammation [39]. These findings were recently confirmed by Zou et al. who showed that human UC-MSC-Exos decreased synthesis of inflammatory cytokines (IFN-*γ*, IL-6, IL-2, IL-17, and TNF-*α*) and increased expression of Foxp3, IL-10, and TGF-*β* in T cells, enhancing their immunosuppressive properties [41]. By inducing expansion of Tregs and by suppressing proliferation of Th17 cells, UC-MSCs-Exos partially restored salivary secretion, downregulated production of autoantibodies, improved intestinal homeostasis, and inhibited infiltration of inflammatory cells in the intestine and submandibular glands of experimental mice [41].

**Figure 2:**
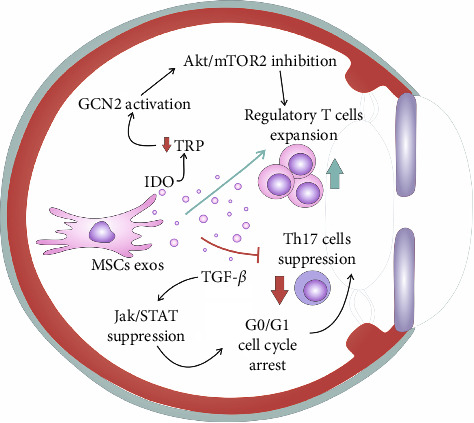
Mesenchymal stem cell–derived exosomes (MSC-Exos)–based regulation of T–regulatory cells (Tregs):Th17 cell ratio in inflamed eyes. MSC-Exos, in indoleamine 2, 3-dioxygenase (IDO)–dependent manner, promote expansion of immunosuppressive Tregs in inflamed eyes of patients with dry eye disease (DED). IDO, derived from MSC-Exos and tolerogenic dendritic cells (DCs), attenuates concentration of tryptophan (TRP) in inflamed microenvironment. Low levels of TRP induce activation of general control non-derepressible 2 (GCN2) kinase which inhibits protein kinase B (PKB/Akt)/mammalian target of rapamycin (mTOR)2 signaling in Tregs and prevents their transdifferentiation in inflammatory Th17 cells. MSC-Exos–derived transforming growth factor beta (TGF-*β*) suppresses activation of Janus kinase/signal transducers and activators of transcription (Jak/Stat) signaling pathway in IL-17-producing Th17 cells, causing G0/G1 cell cycle arrest.

Li et al. demonstrated that intravenous administration of LG-MSC-Exos (at a dose of 50 *μ*g, three times per week for 2 weeks) significantly enhanced the saliva flow rate in experimental mice [37]. Importantly, the number and area of lymphocyte infiltration foci were notably reduced in the salivary glands of LG-MSC-Exos–treated animals compared to those treated with PBS. LG-MSC-Exos downregulated serum levels of Th17-related inflammatory cytokines (IL-6 and IL-17), increased the levels of immunosuppressive TGF-*β*, decreased the presence of Th17 cells, and promoted the expansion of Tregs in the eyes of pSS experimental mice [37]. It is well known that MSC-Exos–sourced IL-10 induces the development of tolerogenic DCs, which then interact with naïve CD4+T cells to drive their differentiation into FoxP3+Tregs, fostering an immunosuppressive environment in the inflamed eyes of pSS patients [25, 26]. Furthermore, MSC-Exos, in a TGF-*β*-dependent manner, hinder the proliferation and expansion of inflammatory Th17 cells [25]. TGF-*β* derived from MSC-Exos inhibits the activation of the Jak-Stat signaling pathway in IL-17–producing Th17 cells, resulting in the cell cycle arrest at the G0/G1 phase [25, 26]. Consequently, LG-MSC-Exos increased the Treg: Th17 ratio in inflamed eyes, leading to the mitigation of ongoing inflammation and the relief of DED-related symptoms (Figure 2) [37].

Similar findings were observed in vitro, after flow cytometry analysis of pSS patients' MNCs which were cultured with LG-MSC-Exos for 72 h [37]. A significant increase in the percentage of CD4+, CD25+, FoxP3+, and Tregs and a decrease in the percentage of Th17 lymphocytes were noted in the population of MNCs from pSS patients exposed to LG-MSC-Exos. In addition, LG-MSC-Exos influenced the secretory profile of T cells, leading to reduced production of inflammatory cytokines (IL-17A, IFN-*γ*, IL-6, and TNF-*α*) and elevated the production of immunosuppressive TGF-*β* and IL-10, confirming therapeutic potential of LG-MSC-Exos in the attenuation of T-cell–driven eye inflammation [37].

Almost identical findings were recently reported by Ma et al. who investigated the therapeutic potential of MSC-Exos which were isolated from the UC (UC-MSC-Exos) [38]. They showed that UC-MSC-Exos inhibited the proliferation of pSS patients' Th17 cells by causing cell cycle arrest at the G0/G1 phase. In addition, UC-MSC-Exos promoted the expansion of Tregs in lymph nodes by increasing the expression of Foxp3 in naïve CD4+T cells [38]. The rise in the Tregs: Th17 ratio was associated with the decreased production of inflammatory cytokines (IFN-*γ*, TNF-*α*, IL-6, IL-17A, and IL-17F) and increased secretion of immunosuppressive TGF-*β* and IL-10 in UC-MSC–Exos-primed T cells primed with MSC-Exos [38].

Based on these promising results, clinical trial (NCT04213248), which is going to investigate the therapeutic potential of UC-MSC-Exos in the alleviation of DED-related symptoms in patients suffering from oGVHD, is currently recruiting patients. According to the study protocol, patients with oGVHD will be given artificial tears for 14 days to establish a baseline, after which they will receive UC-MSC-Exos eye drops (10 μg/drop; four times a day) for two weeks. Monitoring over the 12-week follow-up period will include assessments of the ocular surface disease index, conjunctiva redness scores, tear secretion, tear break time, ocular surface staining, best corrected visual acuity (BCVA), and tear meniscus height. The initial findings from this trial are anticipated within the next 2 years.

Exos will be isolated from the blood samples of healthy and DED patients in order to identify small RNA molecules which are differentially expressed in DED patients and to identify their role in the development and progression of DED.

Patients will receive artificial tears for 2 weeks, followed by PSC-MSC-Exos–containing eye drops (0.125 mL/single eye/four time a day) for 12 weeks. Changes in Ocular Surface Index Score (OSDI), tear film breakup time, Ocular Surface Staining Score (OSSC), tear meniscus height, and BCVA will be analyzed to determine effectiveness of PSC-MSC-Exos in DED treatment.

Another clinical trial (NCT06543667), which is currently recruiting patients, will investigate therapeutic potential of limbal stem cell–derived exosomes (LSC-Exos) in the attenuation of DED-related symptoms in patients who did not optimally respond to tear drops–based conventional treatment. Patients with moderate or severe DED will receive artificial tears for 2 weeks and then LSC-Exos (10 *μ*g/drop; 0.15 mL/single eye/one time), four times a day for three months. The efficacy of LSC-Exos–based therapy will be assessed by measuring: (i) OSDI, (ii) concentration of inflammatory cytokines (IL-1, IL-6, IL-8, TNF-*α*, and IFN-*γ*) in patients' tears, (iii) tear secretion amount, (iv) tear film breakup time, (v) ocular redness, (vi) tear meniscus, and (vii) BCVA. This study will recruit 30 DED patients and should start in January 2025.

## 5. Therapeutic Potential of MSC-Exos in the Suppression of Severe B-Cell–Driven Eye Inflammation

MSC-Exos are enriched with MSC-derived miRNAs, small noncoding RNA molecules that regulate gene expression by affecting stability of mRNAs and by impairing protein synthesis in eye-infiltrated immune cells, modulating their phenotype and function [42]. Xing et al. recently demonstrated that MSC-sourced miRNA-125b was mainly responsible for the immunosuppressive effects of MSC-Exos in the attenuation of DED-related symptoms [43]. LG-MSC-Exos (50 μg/mouse) administered intravenously (3 times per week for 2 weeks) in mice with spontaneous pSS, effectively suppressed the harmful inflammatory reaction in the inflamed lacrimal and salivary glands, completely relieving DED-related symptoms [43]. The treatment with LG-MSC-Exos led to a significant decrease in the total number of auto-antibody–producing CD19-CD138+ plasma cells in the spleens of the experimental animals. Likewise, a notably reduced percentage of CD19+ CD20-CD27+ CD38+ plasma cells was observed in the LG-MSC-Exos–primed MNCs previously isolated from pSS patients [43]. Importantly, the analysis of mRNA expression in LG-MSC-Exos–treated B-lymphocytes indicated that the LG-MSC-Exo–derived miRNA-125b played a key role in modulating auto-antibody production and in dampening the B-cell–mediated autoimmune response in pSS [43].

The transcript of PR domain zinc finger Protein 1 (PRDM1) gene (also known as blimp) was the main intranuclear target of MSC-derived miRNA-125b [43]. MSC-sourced miRNA-125b recognized specific sequences in the mRNA called miRNA recognition elements (MREs) or binding sites. The binding between the MSC-sourced miRNA-125b and PRDM1 mRNA was mediated by the RNA-induced silencing complex (RISC) and argonaute proteins [42]. The binding of the MSC-derived miRNA-125b to targeted PRDM1 mRNA triggered its degradation by promoting the recruitment and activation of exonuclease complex. In addition, MSC-derived miRNA-125b prevented the interaction between PRDM1 mRNA and ribosomes, impairing the synthesis of PRDM1 protein in plasma cells [42]. PRDM1 protein is a transcription factor which has been identified as a key regulator of B-cell differentiation into antibody-producing plasma cells [44]. In addition, PRDM1 is known to regulate the expression of various genes involved in immune responses and inflammation. It can modulate the production of proinflammatory cytokines in LG-infiltrated B cells (IL-6 and TNF-*α*), importantly contributing to the inflammatory processes in DED. PRDM1 can also influence the expression of B-cell–recruiting chemokines (CXCL13) and adhesion molecules (E and P selectins), which are involved in the recruitment and activation of B cells at the ocular surface [43]. Furthermore, PRDM1 has been found to interact with the STAT-3 transcription factor which regulates the development of B cells from their progenitors in bone marrow [43]. Therefore, by inhibiting synthesis of PRDM1, MSC-derived miRNA-125b regulated development, recruitment, and activation of B cells and impaired their differentiation in auto-antibody–secreting plasma cells, attenuating detrimental B-cell–driven immune response in the inflamed eyes of DED patients [42].

## 6. MSC-Exo–Based Therapy of Severe DED: Current Challenges and Future Perspectives

Since MSC-Exos may deliver their cargo directly in the target cells, these EVs may be used as vehicles for the transport of bioactive molecules. Ma et al. recently developed eye drops composed of ascorbic acid (AA) and MSC-Exos (mExo@AA) [45]. These eye drops were prepared based on the capacity of AA to reduce ROS production of eye-infiltrated immune cells and MSC-Exos to deliver their cargo directly in target cells [45]. In vitro results showed that mExo@AA improved viability and migration of injured CECs, attenuated production of inflammatory cytokines (IL-1*β* and IL-6), and induced generation of immunosuppressive phenotype in macrophages [45]. By using the murine model of BAC-induced eye inflammation, Ma et al. demonstrated that mExo@AA inhibited ROS production in eye-infiltrated immune cells, attenuated detrimental immune response in inflamed eyes, enhanced repair and regeneration of ocular surface barrier, and efficiently restored tear production in experimental animals. Importantly, side effects were not observed after topical application of mExo@AA, indicating their potential clinical use [45].

Despite the fact that results from a large number of preclinical studies indicated therapeutic potential of MSC-Exos in the modulation of immune cell–driven eye inflammation and DED treatment [46], it should be noted that several challenges still limit their clinical application [47, 48]. There is a lack of standardized protocols for the isolation and characterization of MSC-Exos. Different isolation methods and techniques can yield MSC-Exos with varying purity, size, and content [47]. Establishing standardized procedures for isolation and characterization is essential to ensure consistent and reproducible results. Also, MSC-Exos has to be produced in large quantities for clinical applications [47]. However, current methods for their production and purification are often time-consuming, expensive, and yield low quantities. Developing scalable and cost-effective production methods is crucial to meet the demands of clinical use [48].

Although MSCs which reside in different organs share a large number of morphological and functional characteristics, MSCs are not homogenous cell populations [16, 17, 49]. Therefore, the content of MSC-Exos, isolated from diverse tissue sources, can vary depending on the phenotype, function, and tissue source of their parental MSCs [25, 26]. This heterogeneity can lead to inconsistent therapeutic effects and challenges in defining a specific set of functional characteristics for clinical use [25, 26]. Moreover, MSC-Exos are sensitive to environmental conditions, such as temperature, freeze-thaw cycles, and storage duration [47, 48]. Maintaining the stability and integrity of exosomes during storage and transportation is critical for their clinical application [47, 48]. Identifying and standardizing specific markers or cargo profiles associated with therapeutic efficacy and developing appropriate storage conditions and techniques, such as cryopreservation, is necessary to preserve the functional properties and therapeutic efficacy of MSC-Exos [47, 48].

## 7. Conclusions

Therapeutic potential of MSC-Exos in the treatment of severe DED is relied on their capacity to selectively deliver their cargo directly into the target eye-infiltrated immune cells and ECs, affecting their phenotype and function. MSC-Exos–sourced immunoregulatory proteins and miRNAs may induce generation of anti-inflammatory and immunosuppressive phenotype in eye-infiltrated macrophages, DCs, and T cells, attenuating ongoing inflammation. MSC-Exos created immunosuppressive microenvironment in inflamed eyes by inducing expansion of tolerogenic DCs, M2 macrophages, and Tregs and by suppressing proliferation and effector function of inflammatory DCs, M1 macrophages, and Th1 and Th17 lymphocytes. In addition, by delivering growth factors and antiapoptotic miRNAs in injured goblet cells, acinar cells, and SGECs, MSC-Exos may prevent their apoptosis and could restore impaired function of lacrimal and salivary glands. However, having in mind that MSC-Exos also contain proinflammatory and proapoptotic proteins and nucleic acids, safety profile of each MSC-Exos–based eye drops has to be thoroughly evaluated in clinical trials before these immunoregulatory therapeutic agents could be offered as new remedies for the treatment of severe DED.

## Data Availability

The data that are discussed in this article are presented in the cited studies.
